# Supplementary material for: Sulfated endospermic nanocellulose crystals prevent the transmission of SARS-CoV-2 and HIV-1
Source: Sci Rep. 2023 Apr 28;13:6959. doi: 10.1038/s41598-023-33686-y (PMC10141831; doi:10.1038/s41598-023-33686-y)
Supplement: Supplementary file 1 — Supplementary Information. [file 41598_2023_33686_MOESM1_ESM.docx]

**Supplemental Figures**

**Sulfated endospermic nanocellulose crystals prevent the transmission of SARS-CoV-2 and HIV-1**; Enrique Javier Carvajal Barriga, et al.


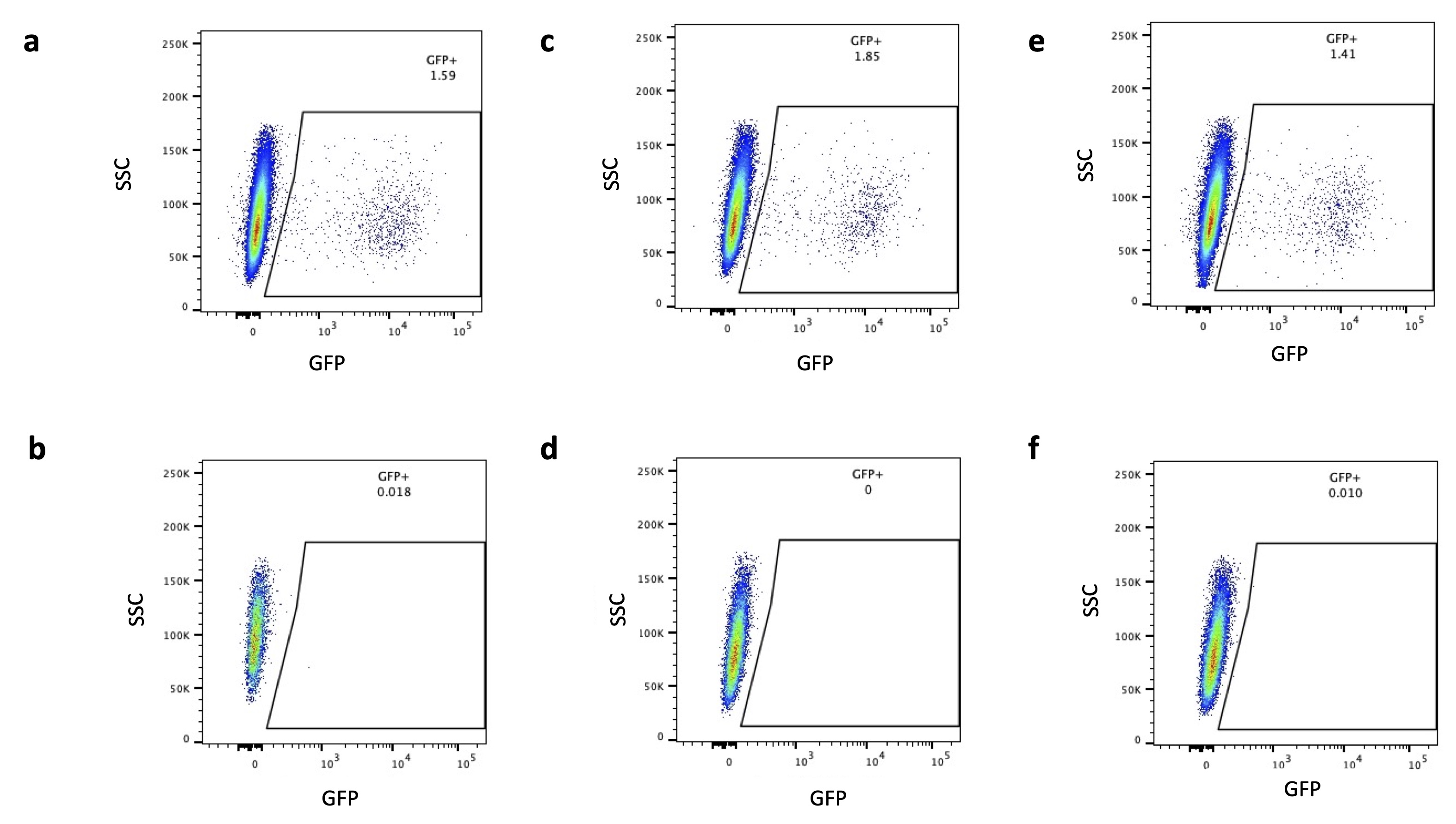


**Supplemental Figure 1. ENC prevented SAR-CoV-2 pseudovirus infection of 293T-ACE2 cells as measured by flow cytometry.**

293T-ACE2 cells were inoculated with SARS-CoV-2 pseudovirus (pv) with a GFP reporter in conditions with and without endospermic nanocellulose (ENC) and molecular lures (ACE2 or anti-SARS-CoV-2 S IgG (IgG)) and incubated for 3 days. Cells were harvested for flow cytometry analysis and % of GFP infected cells was determined for each condition (n=16, one representative experiment is shown). **a.** cells+pv (positive control) **b.** cells+(ENC+pv), **c.** cells+(ACE2+pv), **d.** cells+(ENC+ACE2+pv), **e.** cells+(IgG+pv), **f.** cells+(ENC+IgG+pv).


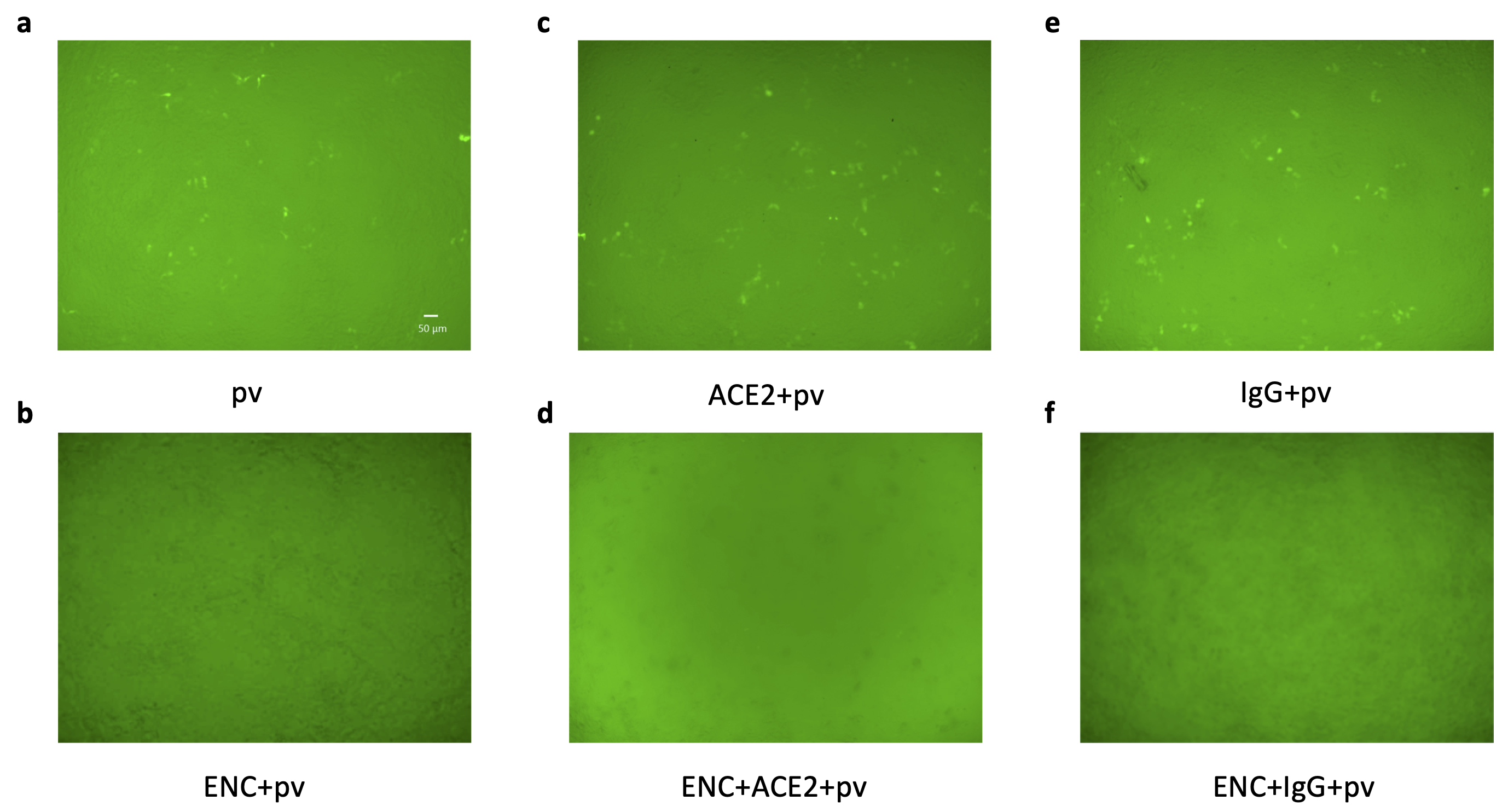


**Supplemental Figure 2. ENC prevented SAR-CoV-2 pseudovirus infection of 293T-ACE2 cells visualized by fluorescent microscopy.**

293T-ACE2 cells were inoculated with SARS-CoV-2 pseudovirus (pv) with a GFP reporter in conditions with and without ENC and molecular lures (ACE2 or anti-SARS-CoV-2 S IgG (IgG)) and incubated for 3 days. Cells were imaged on a fluorescent microscope and GFP+ infected cells were visualized for each condition (n=4, one representative experiment is shown). **a.** cells+pv (positive control) **b.** cells+(ENC+pv), **c.** cells+(ACE2+pv), **d.** cells+(ENC+ACE2+pv), **e.** cells+(IgG+pv), **f.** cells+(ENC+IgG+pv).
